# Prevalence and Determinants of Undiagnosed Diabetes Mellitus Among Adults in Zakho City, Kurdistan Region, Iraq: A Community-Based Cross-Sectional Study

**DOI:** 10.7759/cureus.77618

**Published:** 2025-01-18

**Authors:** Brisik Rashad, Nawfal R Hussein, Vindad Hashim Dirbas, Ibrahim A Naqid

**Affiliations:** 1 Department of Biomedical Sciences, College of Medicine, University of Zakho, Zakho, IRQ; 2 Department of Clinical Sciences, College of Medicine, University of Zakho, Zakho, IRQ

**Keywords:** community, diabetes mellitus, prevalence, undiagnosed, zakho city

## Abstract

Objective

This study aimed to study the prevalence of undiagnosed diabetes mellitus (DM) and its associated factors in adults aged over 18 in Zakho City, Kurdistan Region, Iraq.

Methods

A cross-sectional study was conducted recruiting 537 participants with a mean age of 35.43±13.88 years. Data on sociodemographic characteristics, health behaviors, clinical history, and anthropometric measurements, including body mass index (BMI) and waist circumference, were collected.

Results

The study sample comprised 345 (64.2%) men and 192 (35.8%) women, with 324 (60.3%) being married and 246 (45.8%) having a university-level education or higher. Regarding health behaviors, 218 (40.6%) participants engaged in physical activity, 180 (33.5%) were smokers, and 196 (36.5%) reported sleep disturbances, averaging 7.10±2.02 hours per night. Anthropometric assessments revealed that 209 (38.9%) were overweight, 131 (24.4%) were obese, and 273 (50.8%) had high waist circumference. The prevalence of abnormal glucose levels was 51 (9.5%) (95% CI: 7.02-11.98) including 14 (2.6%) (95% CI: 1.26-3.95) with undiagnosed DM and 37 (6.9%) (95% CI: 4.75-9.03) with prediabetes mellitus (pre-DM). Multivariate analysis showed significant associations between abnormal glucose levels and increased age (p<0.0001), high BMI (≥25 kg/m²) (p<0.0001), and high waist circumference (p<0.0001). Specifically, age was a significant predictor for pre-DM (adjusted odds ratio (aOR) 1.04; 95% CI: 1.01-1.07), while age and high waist circumference predicted undiagnosed DM (aOR 1.90; 95% CI: 1.03-1.16).

Conclusion

This study provided the first estimate of undiagnosed DM in the Kurdistan Region. The current study showed that the prevalence of undiagnosed DM and impaired fasting glucose were significant emphasizing age, BMI, and waist circumference as significant risk factors. These findings suggest the need for targeted screening and preventive strategies in this population to improve DM detection and management.

## Introduction

Diabetes mellitus (DM) is increasingly becoming widespread globally. The International Diabetes Federation (IDF) claims that in 2021, there were about 537 million adults with DM (10.5% of the global adult population). Even worse is the fact that out of these victims, approximately 240 million or 44.7% are ignorant of their condition. Approximately 90% of all cases belong to type 2 diabetes mellitus (T2DM). The burden of DM is expected to rise, reaching 643 million by 2030 (11.3%) and 783 million (12.2%) by 2045 [1].

Alongside DM, the term "prediabetes mellitus (pre-DM)" is used for individuals whose blood glucose is too high to be regarded as normal (fasting blood glucose >100 mg/dl and <126 mg/dl or two hours postprandial glucose >140 mg/dl and less than 200 mg/dl), but doesn't meet the criteria to be categorized as DM [2]. The global prevalence of pre-DM including impaired fasting glucose and impaired glucose tolerance and/or HbA1C 5.7-6.4% (39-47 mmol/mol) is also increasing. In 2021, it was estimated that about 860 million adults representing approximately 16.8% of the global population aged over 18 years were affected by pre-DM according to the IDF [1].

Undiagnosed DM refers to someone with DM who has not been diagnosed by a physician but has a blood glucose level meeting the established criteria for the diagnosis of DM [3]. According to the most recent study done on the prevalence of undiagnosed DM in the total population of the United States, 2.7% of adults aged 20 or above have undiagnosed DM, compared to 5.1% for diagnosed DM [4]. Undiagnosed DM can only be determined by a health survey, in which individuals' blood glucose levels are tested and they are asked whether they have been diagnosed with DM or not through their past medical history [3]. Studies have shown that patients may remain in the asymptomatic phase of pre-DM and T2DM for about 5-6 years before being diagnosed, during which time complications of DM may develop [5,6]. According to a study done by the IDF in 2021, the highest proportion of undiagnosed DM was in Africa, Western Pacific (52.8%), and Southeast Asia regions (51.3%), respectively, with the lowest proportion being in North America and the Caribbean region (24.2%) [5].

The coronavirus disease 2019 (COVID-19) and other pandemics had a deleterious impact on the healthcare system in Iraq [7-10]. The pandemic may have impacted DM prevalence in the community by increasing sedentary behaviors, stress levels, and changes in dietary habits especially among those with limited access to healthcare and regular screenings during lockdowns and healthcare disruptions. Few studies have been conducted in the Kurdistan Region of Iraq about DM [11-13]. However, no community-based study has been conducted to investigate the prevalence of undiagnosed DM in the region. This study aimed to examine the prevalence of undiagnosed DM and identify the association between undiagnosed DM and sociodemographic status in individuals aged more than 18 in Zakho City, Kurdistan Region, Iraq.

## Materials and methods

Study design

A community-based cross-sectional study was conducted in Zakho City, Kurdistan Region, Iraq, on January 9, 2024, for a day (from 10 am to 4 pm). The study included participants aged 18 years or older living in Zakho City and meeting the inclusion criteria. We used the multistage sampling method. In the first stage, artificial intelligence (AI) was asked to select six different districts in Zakho City randomly (University Campus, Dream City, Taxe Nasara, Taxe Garamira, Chamishko Camp, and Rekava Ni). In each area, household sampling was performed randomly by a random number generator. The proportion p=0.5 is assumed in sample size calculations when the true population proportion is unknown, and this assumption is made to maximize the sample size. Using the formula for an infinite population, the initial sample size was calculated, and the final sample size was approximately 384. The total number of individuals enrolled in this study was 537 with a mean age±standard deviation of 35.73±13.95. Individuals who were previously diagnosed with DM, pregnant women, and adults under 18 years were excluded.

Data collection and procedure

Data were collected using a pretested structured interviewer-administered questionnaire. The questionnaire contained sociodemographic, behavioral, and clinical characteristics.

Anthropometric data like weight was measured using a high-quality weighing scale device, and height was measured in a standing position. Body mass index (BMI) was calculated by dividing weight by height in a meter square. A BMI of <25 kg/m² was considered normal, 25-29.9 kg/m² overweight, and ≥30 kg/m² obese [14]. Waist circumference was measured by placing a tape at the level between the lowest rib margin and the top of the iliac crest around the body. A waist circumference equal to or less than 94 cm in men and 80 cm in women was classified as normal, while a waist circumference more than 94 cm in men and 80 cm in women was classified as high [15].

Glucose levels were measured using fingerstick glucose devices. Patients were asked whether they ate something or were fasting for several hours to define their glucose levels as random or fasting blood glucose levels, respectively. Fasting blood glucose levels of 126 mg/dL or above were considered as having DM, whereas fasting blood glucose levels of 100-125 mg/dL were regarded as having "impaired fasting glucose" levels. However, anyone with random blood glucose levels of 200 mg/dL or above was classified as diabetic, while those who had random blood glucose levels of 140-199 mg/dL were considered as "impaired fasting blood" cases [2]. Those with fasting glucose levels of ≥100 mg/dL or random glucose levels of ≥140mg/dL were tested again by fingerstick glucose devices. After the confirmation of high glucose levels above normal, laboratory workers took 3-5 ml of blood in those participants to be tested for HbA1C using the high-performance liquid chromatography (HPLC) method.

After the interview, those participants who were diagnosed with DM were informed about their DM status a week after the interview and advised to consult a DM specialist to get help for further treatment. Participants with impaired glucose levels were also informed about DM and its complications and risk factors, such as smoking, physical inactivity, sedentary lifestyle, sleep disturbances, and alcohol consumption, and were advised to see a healthcare facility for further checkups.

Physical activity is defined as any bodily movement generated by the skeletal muscle that requires energy expenditure [16]. Participants who were still smoking during the interview were considered as smokers.

Data analysis

The data were coded, cleaned, entered, and revised using Microsoft Excel (Microsoft Corporation, Redmond, Washington, United States) and then exported to IBM SPSS Statistics for Windows, Version 26.0 (Released 2019; IBM Corp., Armonk, New York, United States) for further analysis. The descriptive statistics including means, medians, percentages, and standard deviations presented in tables and texts were calculated to describe the study population. The chi-squared tests, Fisher's tests, and logistic regression were employed to identify factors significantly associated with pre-DM and undiagnosed DM. Bivariable regression analysis was first performed to calculate the crude odds ratios for each variable. Variables with p-values less than 0.2 were then included in the multivariable logistic regression model. This model was used to identify statistically significant associations between the independent variables and the outcome, as well as to calculate the adjusted odds ratios with 95% confidence intervals. Variables with a p-value of <0.05 were considered statistically significant.

Ethical consideration

Ethical approval was obtained from the Scientific Research and Ethics Committee of the College of Medicine, University of Zakho (approval number: Oct 2023/UoZ e18). Participants were informed about the study and its objectives. Verbal informed consent was obtained from each respondent before the administration of the questionnaire.

## Results

The total number of individuals enrolled in this study was 537 with a mean age±standard deviation of 35.43±13.88 (Table 1). About two-thirds (64.2%; n=345) of the participants were men. Of the study participants, 60.3% (n=324) were married. The majority of the study participants were unemployed (46.9%; n=252), 32.4% (n=174) were government-employed, and 20.7% (n=111) were working privately. Approximately a third of the participants (33.5%; n=180) were smokers. Of the study participants, 35.5% (n=196) reported that they have sleep disturbance. The average sleep duration was 7.10±2.02 hours among the participants (Table 1).

**Table 1:** Sociodemographic and behavioral characteristics of the study participants (n=537) NGM: normal glucose metabolism; AGM: abnormal glucose metabolism; UDDM: undiagnosed diabetes mellitus; pre-DM: prediabetes mellitus; SD: standard deviation *: significant at <0.05

Variables	NGM	AGM		Total, N (%)	P-value
		Pre-DM, N (%)	UDDM, N (%)		
Age (in years)					
Mean±SD	34.73±13.46	45.02±15.35	47.85±8.12	35.43±13.88	<0.0001>
18-24	147 (96.7)	5 (3.3)	0 (0)	152 (28.3)	
25-34	127 (97.7)	3 (2.3)	0 (0)	130 (24.2)	
35-44	110 (85.9)	12 (9.4)	6 (4.7)	128 (23.8)	
45-54	61 (83.6)	7 (9.6)	5 (6.8)	73 (13.6)	
≥55	41 (75.9)	10 (18.5)	3 (5.6)	54 (10.1)	
Sex					
Male	316 (91.6)	20 (5.8)	9 (2.6)	345 (64.2)	0.2475
Female	170 (88.5)	17 (8.9)	5 (2.6)	192 (35.8)	
Educational status					
No formal education	63 (80.8)	10 (12.8)	5 (6.4)	78 (14.5)	0.005
Primary or less	83 (88.3)	8 (8.5)	3 (3.2)	94 (17.5)	
Secondary or less	108 (90.8)	8 (6.7)	3 (2.5)	119 (22.2)	
University	232 (94.3)	11 (4.5)	3 (2.1)	246 (45.8)	
Marital status					
Single	204 (95.8)	8 (3.8)	1 (0.5)	213 (39.7)	0.001
Married	282 (87)	29 (9)	13 (4)	324 (60.3)	
Occupation					
Unemployed	227 (90.1)	17 (6.7)	8 (3.2)	252 (46.9)	0.95
Government employed	158 (90.8)	12 (6.9)	4 (2.3)	174 (32.4)	
Private employed	101 (91)	8 (7.2)	2 (1.8)	111 (20.7)	
Current smoking					
Yes	161 (89.4)	12 (6.7)	7 (3.9)	180 (33.5)	0.54
No	325 (91)	25 (7)	7 (2)	357 (66.5)	
Physical activity (minutes/week)					
Yes	203 (93.1)	10 (4.6)	5 (2.3)	218 (40.6)	0.087
No	283 (88.7)	27 (8.5)	9 (2.8)	319 (59.4)	
Sleep disturbance					
Yes	181 (92.3)	12 (6.1)	3 (1.5)	196 (36.5)	0.27
No	305 (89.4)	25 (7.3)	11 (3.2)	341 (63.5)	
Sleep duration (mean±SD)	6.83±1.82	7.33±2.09	7.15±2.17	7.10±2.02	0.092

The anthropometric measurements and BMI results of participants showed that the rates of those who are overweight, of normal weight, obese, and underweight were 38.9% (n=209), 33.5% (n=180), 24.4% (n=131), and 3.2% (n=17), respectively. The waist circumference of approximately half (50.8%; n=273) of the participants was high, with the result of high-risk central obesity being 31.65% (n=170). Family history of DM was 40.4% (n=217) positive among individuals. About 43.2% (n=232) had checked their blood glucose level previously, and 8.8% (n=47) had checked last month. Previous history of hypertension and dyslipidemia were reported by 9.5% (n=51) and 14% (n=75) of participants, respectively. Also, 3.7% (n=20) of individuals had a past history of ischemic heart disease, and 3% (n=16) had thyroid disease. Of the total participants, 14% had symptoms of DM (Table 2).

**Table 2:** Clinical characteristics of the study participants (n=537) NGM: normal glucose metabolism; AGM: abnormal glucose metabolism; UDDM: undiagnosed diabetes mellitus; pre-DM: prediabetes mellitus; DM: diabetes mellitus; SD: standard deviation *: significant at <0.05

Variables	NGM	AGM		Total, N (%)	P-value
		Pre-DM, N (%)	UDDM, N (%)		
Ever checked blood sugar					
Yes	205 (88.4)	19 (8.2)	8 (3.4)	232 (43.2)	0.14
No	281 (92.1)	18 (5.9)	6 (2)	305 (56.8)	
Checked blood sugar last month					
Yes	44 (93.6)	1 (2.1)	2 (4.3)	47 (8.8)	0.45
No	442 (90.2)	36 (7.3)	12 (2.4)	490 (91.2)	
Family history of DM					
Yes	196 (90.3)	16 (7.4)	5 (2.3)	217 (40.4)	0.26
No	290 (90.6)	21 (6.6)	9 (2.8)	320 (59.6)	
Symptoms of DM					
Yes	65 (86.7)	9 (12)	1 (1.3)	75 (14)	0.22
No	421 (91.1)	28 (6.1)	13 (2.8)	462 (86)	
History of hypertension					
Yes	43 (84.3)	5 (9.8)	3 (5.9)	51 (9.5)	0.13
No	443 (91.2)	32 (6.6)	11 (2.3)	486 (90.5)	
History of dyslipidemia					
Yes	63 (84)	8 (10.7)	4 (5.3)	75 (14)	0.038
No	423 (91.6)	29 (6.3)	10 (2.2)	462 (86)	
History of ischemic heart disease					
Yes	16 (80)	4 (20)	0 (0)	20 (3.7)	0.1
No	470 (90.9)	33 (6.4)	14 (2.7)	517 (96.3)	
History of thyroid disease					
Yes	14 (87.5)	2 (12.5)	0 (0)	16 (3)	0.68
No	472 (90.6)	35 (6.7)	14 (2.7)	521 (97)	
Body mass index (kg/m^2^)					
Mean±SD	27.38±9.10	32.72±18.34	33.68±5.46	27.76±10.12	<0.001
Underweight (<18.5)	17 (100)	0 (0)	0 (0)	17 (3.2)	
Normal (18.5-24.9)	174 (96.7)	6 (3.3)	0 (0)	180 (33.5)	
Overweight (25-29.9)	192 (91.9)	14 (6.7)	3 (1.4)	209 (38.9)	
Obese (≥30)	103 (78.6)	17 (13)	11 (8.4)	131 (24.4)	
Waist circumference (cm)					
Mean±SD	90.01±14.25	99.13±16.39	105.71±9.35	90.58±14.57	<0.001
Normal	254 (96.2)	9 (3.4)	1 (0.4)	264 (49.2)	
High	232 (85)	28 (10.3)	13 (4.8)	273 (50.8)	

Prevalence of pre-DM and undiagnosed DM

The overall prevalence of abnormal glucose levels was 9.5% (n=51) (95% CI: 7.02-11.98), with undiagnosed DM being 2.6% (n=14) (95% CI: 1.26-3.95) and pre-DM 6.9% (n=37) (95% CI: 4.75-9.03). The prevalence of pre-DM and undiagnosed DM were higher in men (3.72% (men) versus 3.16% (women) for pre-DM and 1.67% (men) versus 0.93% (women) for DM) (Table 1).

Factors associated with blood glucose levels

Factors Associated With Abnormal Blood Glucose Levels

Among those with abnormal glucose levels (a total of 51 individuals), age, educational status, marital status, concomitant dyslipidemia, high BMI, and high waist circumference were found to be significantly and independently related to the abnormal blood glucose levels (Table 1 and Table 2). In multivariate logistic regression, age, high waist circumference, and BMI ≥25 kg/m² were significantly associated with abnormal blood glucose levels with adjusted odds ratios (aORs) of 1.03 (95% CI: 1.00-1.05), 1.03 (95% CI: 1.01-1.06), and 2.75 (95% CI: 1.06-7.15), respectively.

Factors Associated With Pre-DM

Among those diagnosed with pre-DM (a total of 37 individuals), in univariate analysis, age, marital status, educational status, history of ischemic heart disease, waist circumference, and BMI were significantly associated with an increased prevalence of pre-DM (Table 3). In multivariate analysis, only age was found to be significantly associated with pre-DM with an aOR of 1.04 (95% CI: 1.01-1.07) (Table 3).

**Table 3:** Risk factors associated with undiagnosed pre-DM (n=537) pre-DM: prediabetes mellitus; DM: diabetes mellitus; COR: crude odds ratio; aOR: adjusted odds ratio *: significant at <0.05; 1**: remains the same for reference variables in order to measure the p-value of other variables

Variables	Univariate	P-value	Multivariate	P-value
	COR (95% CI)		aOR (95% CI)	
Age (per year)	1.05 (1.02, 1.10)	<0.0001	1.04 (1.01, 1.07)	0.004
Male sex (ref=F)	0.63 (0.32, 1.24)	0.183	0.61 (0.21, 1.27)	0.153
Marital status (ref=single)	2.62 (1.17, 5.85)	0.019	1.01 (0.37, 2.78)	0.981
Educational status				
No formal education	1**		1**	
Primary or less	0.62 (0.23, 1.63)	0.321	0.64 (0.21, 1.95)	0.438
Secondary or less	0.47 (0.17, 1.24)	0.128	0.67 (0.21, 2.10)	0.488
University	0.30 (0.12, 0.73)	0.009	0.63 (0.19, 2.10)	0.446
Occupation				
Unemployed	1**		1**	
Government employed	1.01 (0.47, 2.18)	0.97	1.26 (0.46, 3.45)	0.647
Private employed	1.06 (0.44, 2.53)	0.9	1.70 (0.56, 5.01)	0.341
Current smoking (ref=non-smoker)	0.97 (0.48, 1.98)	0.931	0.77 (0.32, 1.87)	0.576
Physical activity (ref=no physical activity)	1.94 (0.92, 4.10)	0.083	1.50 (0.65, 3.46)	0.342
Sleep disturbance	0.81 (0.39, 1.65)	0.559	0.63 (0.29, 1.4)	0.263
Ever checked blood sugar (ref=no)	0.691 (0.35, 1.35)	0.083	0.88 (0.40, 1.94)	0.755
Checked blood sugar last month (ref=no)	3.58 (0.48, 26.77)	0.213	6.81 (0.80, 58.11)	0.079
Family history of DM	1.12 (0.57, 2.21)	0.728	1.03 (0.49, 2.15)	0.936
Symptoms of DM	2.08 (0.94, 2.61)	0.071	1.91 (0.77, 4.72)	0.164
History of hypertension	1.61 (0.60, 4.35)	0.347	0.97 (0.31, 3.07)	0.966
History of dyslipidemia	1.85 (0.81, 4.23)	0.144	1.29 (0.50, 3.25)	0.707
History of ischemic heart disease	1.93 (0.42, 8.82)	0.031	2.10 (0.52, 8.2)	0.3
History of thyroid disease	1.93 (0.42, 8.82)	0.398	1.1 (0.21, 5.57)	0.924
Body mass index (kg/m^2^)	3.34 (1.37, 8.17)	0.008	1.04 (1.01, 1.07)	0.067
Waist circumference (cm) (per cm)	1.04 (1.02, 1.06)	0.001	1.01 (0.98, 1.04)	0.479

Factors Associated With Undiagnosed DM

In univariate logistic regression analysis for undiagnosed DM (a total of 14 individuals), age, marital status, educational level, waist circumference, and BMI were significantly associated with increased DM, while in multivariate analysis, increasing age and high waist circumference were predictors of undiagnosed DM with aORs of 1.04 (95% CI: 1.00-1.08) and 1.90 (95% CI: 1.03-1.16) (Table 4).

**Table 4:** Risk factors associated with undiagnosed DM (n=537) DM: diabetes mellitus; COR: crude odds ratio; aOR: adjusted odds ratio *: significant at <0.05

Variables	Univariate	P-value	Multivariate	P-value
	COR (95% CI)		aOR (95% CI)	
Age (per year)	1.06 (1.02, 1.09)	0.001	1.04 (1.00, 1.08)	0.039
Male sex (ref=F)	0.96 (0.32, 2.93)	0.955	1.33 (0.23, 7.85)	0.748
Marital status (ref=single)	9.40 (1.22, 72.46)	0.031	5.51 (0.43, 69.99)	0.188
Educational status				
No formal education	1		1	
Primary or less	0.45 (0.11, 1.98)	0.294	0.284 (0.05, 1.64)	0.16
Secondary or less	0.35 (0.08, 1.51)	0.015	0.56 (0.81, 5.96)	0.568
University	0.16 (0.038, 0.7)	0.015	0.65 (0.88, 4.77)	0.67
Occupation				
Unemployed	1	1		
Government employed	0.72 (0.21, 2.43)	0.594	0.40 (0.06, 2.38)	0.314
Private employed	0.56 (0.12, 2.69)	0.471	0.44 (0.06, 3.38)	0.426
Current smoking (ref=non-smoker)	2.02 (0.70, 5.85)	0.196	3.25 (0.80, 13.23)	0.1
Physical activity (ref=yes)	1.29 (0.43, 3.91)	0.651	0.54 (0.12, 2.38)	0.418
Sleep disturbance	0.46 (0.13, 1.67)	0.237	0.29 (0.06, 1.30)	0.106
Ever checked blood sugar	0.55 (0.18, 1.60)	0.271	0.71 (0.17, 2.97)	0.644
Checked blood sugar last month	0.80 (0.13, 2.75)	0.509	1.60 (0.21, 12.35)	0.651
Family history of DM	0.82 (0.27, 2.50)	0.729	0.76 (0.20, 2.90)	0.689
Symptoms of DM	0.50 (0.06, 3.87)	0.505		
History of hypertension	2.81 (0.75, 10.46)	0.123	0.96 (0.13, 5.28)	0.959
History of dyslipidemia	2.68 (0.82, 8,82)	0.104		
History of ischemic heart disease	-	-	-	-
History of thyroid disease	-	-	-	-
Body mass index (kg/m^2^) (per unit)	1.029 (1.00, 1.057)	0.034	1.02 (0.97, 1.07)	0.408
Waist circumference (per cm) (ref=normal)	1.05 (1.03, 1.11)	<0.0001	1.9 (1.03, 1.16)	0.003

## Discussion

This community-based cross-sectional study aimed to reveal the prevalence of pre-DM and undiagnosed DM and associated factors among adults ≥18 years old in Zakho City.

The estimated prevalence of pre-DM in the current study was 6.9%, which is lower than the rates reported in France (7.2%), Iran (12%), and Ethiopia (10.5%) [17-19]. However, this prevalence is higher than the rates in a study done in Swaziland (6.5%) [20]. Pre-DM is an intermediate state of raised blood glucose between normal glucose tolerance and T2DM. People with pre-DM carry an increased risk of T2DM, with approximately 5-10% of people developing T2DM each year. Previous studies issued that pre-DM is also associated with DM complications like nephropathy, small fiber neuropathy, diabetic retinopathy, and an increased risk of macrovascular disease [21]. Age, marital status, educational status, history of ischemic heart disease, waist circumference, and BMI were found to be significantly associated with pre-DM in the present study. Our study showed that there's a significant relationship between age and the development of pre-DM in both univariate and multivariate analyses, consistent with previous reports [17,18,20,22,23]. The prevalence of pre-DM was the highest among the age group of 35-44 years. Consistent with the present study, BMI is regarded as a strong risk factor for pre-DM [24,25]. Previous reports have shown that an increase in BMI is significantly associated with the progression of pre-DM to DM [26]. In a study conducted in China, it was shown that BMI was independently associated with regression to normoglycemia in adults with impaired fasting glucose. Participants with higher BMI had a significantly lower reversal rate than those with a lower BMI [27]. Two studies done in Germany and Singapore showed that weight loss was associated with regression from pre-DM to normoglycemia [28,29].

In contrast to previous studies [20,23,30], we didn't find any significant association between hypertension and pre-DM maybe due to the small sample size of pre-DM. In the present study, we also found a relationship between ischemic heart disease and pre-DM. Numerous studies showed that pre-DM can lead to cardiovascular disease and several mechanisms such as inflammation and vasoconstriction, which can promote atherosclerosis in the coronary arteries, were observed in the pre-DM state [31,32].

The overall prevalence of undiagnosed DM in this study was 2.6%. This is comparable to studies done in the United States (2.6%), Iran (3.8%), and Turkey (3.6%) [33-35]. However, our study findings were lower than the study results conducted in Morocco (5.9%), Kuwait (6.9%), Iraq (11%), and Malaysia (8.9%) [36-39]. The possible explanation for this could be associated with sociodemographic characteristics, lifestyles, and health-seeking behavior. The differences in the prevalence of undiagnosed DM among different studies could be due to variations in sociocultural characteristics, lifestyles, the composition of the study population, differences in diagnostic criteria, and the level of community awareness related to DM and other non-communicable diseases. 

In the present study, we observed that the prevalence of undiagnosed DM increases with increased age. Different previous studies reported that DM increases with advancing age [19,35,36,38-40]. The odds of developing undiagnosed DM increase 1.04 times with increasing age per year. As for gender differences, the prevalence of undiagnosed DM was higher in men than women, but in contrast to previous studies done in Ethiopia and Kuwait, this association didn't reach a significant statistical level [19,37]. According to our study, we found a significant relationship between undiagnosed DM and marital status in univariate analysis (p=0.03), consistent with previous studies from Ethiopia, Turkey, and Malaysia [19,35,39]. On the contrary, a study done in Brazil observed that the risk of increasing undiagnosed DM increases with single, divorced, or widowed status [41]. This is difficult to explain and more studies are recommended to explore this.

Based on educational level, the present study observed a significant association between undiagnosed DM and educational level, consistent with a study done in Ethiopia [40]. Our data suggests that individuals with higher education levels, particularly those with a university education, are more likely to be in the non-DM category compared to those with less education. This might be explained as follows: individuals with higher education might have more awareness about the disease and may have better access to healthcare facilities. In contrast to previous reports [19,42], we didn't find any significant association between undiagnosed DM and physical activity. This could be explained by the small sample size recruited in this study and the difference in the methodology of the other research.

Similarly, the present study showed that smoking status was not associated with increasing undiagnosed DM. This was consistent with studies done in Ethiopia and India [43,44]. We didn't find any statistically significant association between undiagnosed DM and family history, maybe due to the low number of undiagnosed DM cases. On the contrary, the presence of an association between family history and undiagnosed DM was observed in previous studies done in Ethiopia, Sudan, and India [19,45].

We didn't reveal any relationship between undiagnosed DM and hypertension and cardiovascular disease, inconsistent with previous reports [19,39]. The mechanism between hypertension and DM is complex but includes vascular remodeling and increased body fluid volume caused by insulin resistance-inducing hyperinsulinemia and hyperglycemia, leading to increased peripheral artery resistance [46]. Similarly, in the present study, a statistically significant association was not observed between dyslipidemia and undiagnosed DM. Several studies have concluded that there is a significant relationship between dyslipidemia and DM [35,47]. More studies recruiting a larger sample size are needed to explore such a relationship.

Finally, we observed a significant association between undiagnosed DM and increasing BMI, with the prevalence of overweight and obese people being 63.3% among participants. İncreasing weight circumference and central obesity were also significantly associated with undiagnosed DM. This finding was consistent with previous studies [43,48]. This can be explained as follows: the excess abdominal fat in central obesity facilitates insulin resistance by secreting various substances such as inflammatory cytokines (e.g., TNF-α and IL-6), free fatty acids (FFAs), and adipokines (e.g., adiponectin and resistin), leading to inflammation, glucose intolerance, and hyperglycemia [49].

Strengths and limitations of the study

This study was community-based and involved an appropriate sample size. Another major strength of the study was the fact that blood glucose levels were taken twice in patients with fasting blood sugar of equal or more than 100 mg/dL, or random blood sugar of equal or more than 140 mg/dL, together with the use of HbA1C measurement in order to improve reliability and avoid bias. However, the study has several limitations. The lines between rural and urban areas were blended. Therefore, including each group separately in the study is no longer possible. Another limitation of the population-based study is that the study was subject to recall bias regarding self-reported data like physical activity, smoking status, and social behaviors.

## Conclusions

This study provided the first estimate of undiagnosed DM in the Kurdistan Region. The current study showed that the prevalence of undiagnosed DM and impaired fasting glucose were significant. This suggests the importance of promoting the screening for pre-DM and DM, aiming to detect the disease at an early stage, preventing the progression of pre-DM to DM and its complications, and reducing the economic burden of DM through appropriate early intervention and public awareness projects regarding lifestyle changes and adherence to treatment.
